# Standardized approach to extract candidate outcomes from literature for a standard outcome set: a case- and simulation study

**DOI:** 10.1186/s12874-023-02052-x

**Published:** 2023-11-09

**Authors:** KM Veen, A Joseph, F Sossi, P Blancarte Jaber, E Lansac, E Das-Gupta, S Aktaa, JJM Takkenberg

**Affiliations:** 1 Department of cardiothoracic surgery, Erasmus MC, Rotterdam, The Netherlands; 2 International consortium of Health Outcome Measurement, London, UK; 3 Department of Cardiac Pathology, Pitié-Salpêtrière Hospital, Paris, France; 4 Department of Cardiology, Leeds Teaching Hospitals NHS Trust, Leeds, UK

**Keywords:** Methodology, Outcome measures, Cardiovascular disease, Systematic review, PROM

## Abstract

**Aims:**

Standard outcome sets enable the value-based evaluation of health care delivery. Whereas the attainment of expert opinion has been structured using methods such as the modified-Delphi process, standardized guidelines for extraction of candidate outcomes from literature are lacking. As such, we aimed to describe an approach to obtain a comprehensive list of candidate outcomes for potential inclusion in standard outcome sets.

**Methods:**

This study describes an iterative saturation approach, using randomly selected batches from a systematic literature search to develop a long list of candidate outcomes to evaluate healthcare. This approach can be preceded with an optional benchmark review of relevant registries and Clinical Practice Guidelines and data visualization techniques (e.g. as a WordCloud) to potentially decrease the number of iterations. The development of the International Consortium of Health Outcome Measures Heart valve disease set is used to illustrate the approach. Batch cutoff choices of the iterative saturation approach were validated using data of 1000 simulated cases.

**Results:**

Simulation showed that on average 98% (range 92–100%) saturation is reached using a 100-article batch initially, with 25 articles in the subsequent batches. On average 4.7 repeating rounds (range 1–9) of 25 new articles were necessary to achieve saturation if no outcomes are first identified from a benchmark review or a data visualization.

**Conclusion:**

In this paper a standardized approach is proposed to identify relevant candidate outcomes for a standard outcome set. This approach creates a balance between comprehensiveness and feasibility in conducting literature reviews for the identification of candidate outcomes.

**Supplementary Information:**

The online version contains supplementary material available at 10.1186/s12874-023-02052-x.

## Introduction

Value-based healthcare has emerged in recent years as the primary focus for authorities, decision-makers and the public [1–3]. Medical interventions are evaluated on the basis of their safety, efficacy and cost-effectiveness [4–6]. Whilst randomized controlled trials (RCT) are the gold standard method for such an evaluation, the post-marketing surveillance of clinical outcomes using real-world data is increasingly demanded by regulators and the public [1, 4–6]. To compare different healthcare systems in a meaningful way, standardization of outcome measurement is essential. As such, initiatives like the International Consortium for Health Outcomes Measurement (ICHOM), Core Outcome Measures in Effectiveness Trials (COMET) and OMERACT have been founded to promote outcome standardization [7]. Both ICHOM and COMET recommend (modified)- Delphi processes in stakeholders to reach consensus on included outcomes [8]. Nevertheless, before undertaking the voting process, the candidate outcomes have to be extracted from existing literature and registries (and qualitative methods). The identification of candidate outcomes is a crucial step in the process, because erroneously disregarding outcomes in this step results in exclusion of potentially relevant outcomes prior to the voting process. Systematic reviews or scoping reviews are generally performed to identify candidate outcome measures [9, 10]. However, the methods to do so are not standardized, and the PRIMSA guidelines are not specifically designed for these purposes. Additionally, broad searches yields an overwhelming number of studies, and extracting candidate outcomes from all these studies can be a challenging endeavor. Besides, at a certain point (saturation point), repeated (or similar) outcome measures will be identified, resulting in inefficiency and waste of resources.

In this paper we describe and validate a standardized efficient approach to identify candidate outcomes for the purpose of developing standardized sets and we investigate if this method is able to achieve full saturation using simulated cases, in which the total of candidate outcomes is known. Additionally, we illustrate the utilization of the standardized approach by providing a real-world example.

## Methods

The methodology is based on a iterative saturation approach, which can be preceded by benchmark review and/or data visualization techniques to screen (using frequent words in abstracts) the studies that have been extracted from a systematic search using a scoping search. We illustrate practical use of the methodology in the ICHOM standard set for heart valve disease (HVD). Data saturation techniques to identify candidate outcomes have been proposed and used before, nevertheless in literature several algorithms have been used to achieve saturation, and it is unknown if these algorithms reach full saturation or if several candidate outcomes are missed altogether [7, 11]. This method can be used to both identify outcomes (what is being measured e.g. pain) and outcome measures (how it is being measured e.g. VAS score), and can additionally be used to identify case-mix variables [7]. We will exclusively refer to *outcomes* in the provided examples.

### Iterative saturation approach of literature

A systematic literature review is an essential component in identifying candidate outcomes for a standardized outcome set, also called core outcome set in COMET and OMERACT initiatives. It is recommended to conduct a systematic search in collaboration with a medical information specialist [7]. Multiple medical literature databases should be included. Duplicate articles should be removed before selecting relevant articles. In case of ICHOM, they focus on outcomes (and subsequent outcome measures) of importance to patients, as such PROMs have been incorporated as an integral part of all ICHOM sets. A separate search was done for PROMS specifically, and this yielded a relatively small number of studies that could all be reviewed. In case of clinical outcomes, this searched yielded 17,166 articles. (Table 1).

**Table 1:** Search strategy of the systematic review

Goal	Search terms	Databases	Number of articles
Identifying (clinical) outcomes	See supplementary text 2	Embase	17,166
Identifying PROMs	See supplementary text 3	Embase, MEDLINE OVID®, Web of Science, Cochrane CENTRAL register of Trials	856
Data visualization all abstracts	“Heart Valve Disease”	MEDLINE OVID®	142,279

PROMs: patient-reported outcome measures

**Fig. 1:**
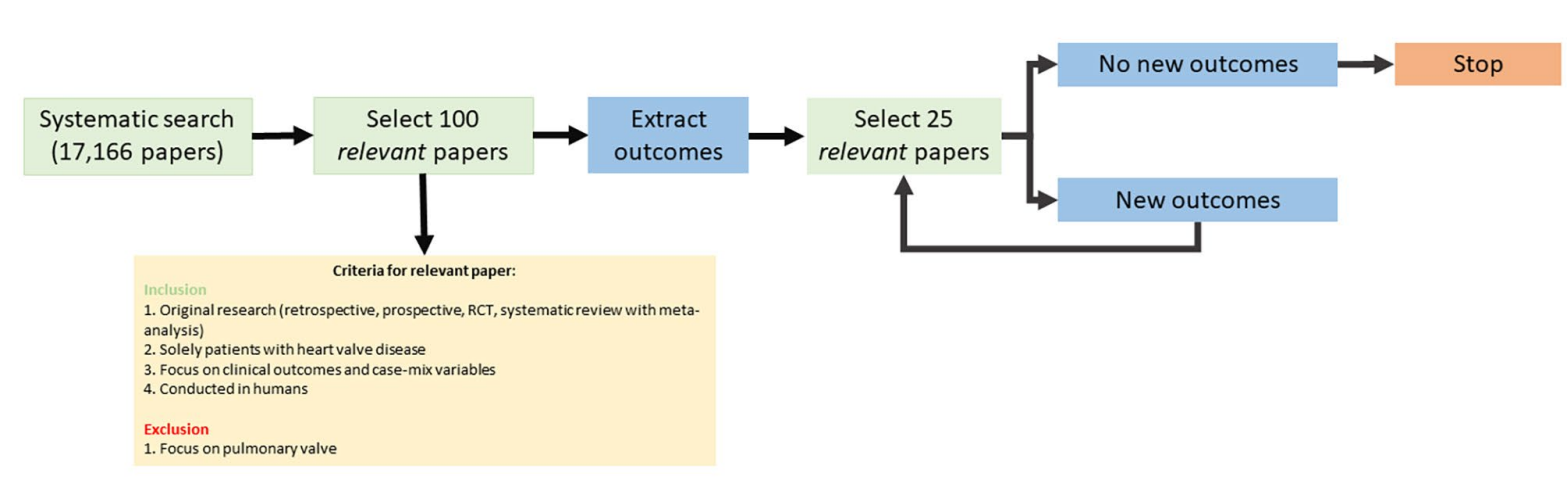
Graphical representation of the iterative algorithm to achieve outcome saturation

To balance the tradeoff between unnecessarily reviewing all these studies and saturation of candidate outcomes (whether all relevant outcomes are identified) an iterative algorithm was used of randomly selecting a batch of articles from an extensive literature search and keep reselecting new batches of articles until saturation of outcomes is achieved. The steps of this algorithm are described in Table 2 and visually represented in Fig. 1. In order to potentially decrease the number of iterations needed to achieve saturation one may perform a benchmark review and data visualization to start with a list of outcomes in the first round of the iteration process.

**Table 2:** Steps of the iterative algorithm to select articles until saturation of all candidate outcomes, with an example of the heart valve disease standardized set

Step	Description	Example HVD set
1	Define research goal	Identify all clinical outcomes used in HVD
2	Define inclusion and exclusion criteria for *relevant* articles	1. Original research (retrospective, prospective, RCT, systematic review with meta-analysis) 2. Solely patients with heart valve disease (excluding the pulmonary valve) 3. Focus on clinical outcomes and case-mix variables 4. Conducted in humans
3	Conduct literature search (Table 1)	Yielded: 17,279 articles
4	Select 100 random articles	Random number generator corresponding to index number articles
5	Extract outcomes from initial randomly selected batch (100 articles)	52 outcomes were identified (including results from benchmark review and data visualisation).
6	Extract outcomes from subsequent randomly selected batch (new 25 articles)	4 new outcomes were identified
7	Repeat step 6 until no new outcomes are identified	In total 150 articles were reviewed

### Benchmark review

In most domains of disease, a wealth of registries and guidelines exist for the capture of clinical outcomes. These resources provide excellent starting points for the identification of candidate outcomes for a standardized set. In the case of the HVD set these resources included several well-known valve registries, as well as international Clinical Practice Guidelines for the management of patients with HVD identified by the stakeholders. Although, the benchmark review is not based on a systematic search and does not capture all outcomes, it provides an overview of several outcomes and way of measuring them. This method is optional and can be performed preceding the iterative saturation approach to reduce the number iterations needed to achieve maximum saturation.

### Data visualization

**Fig. 2:**
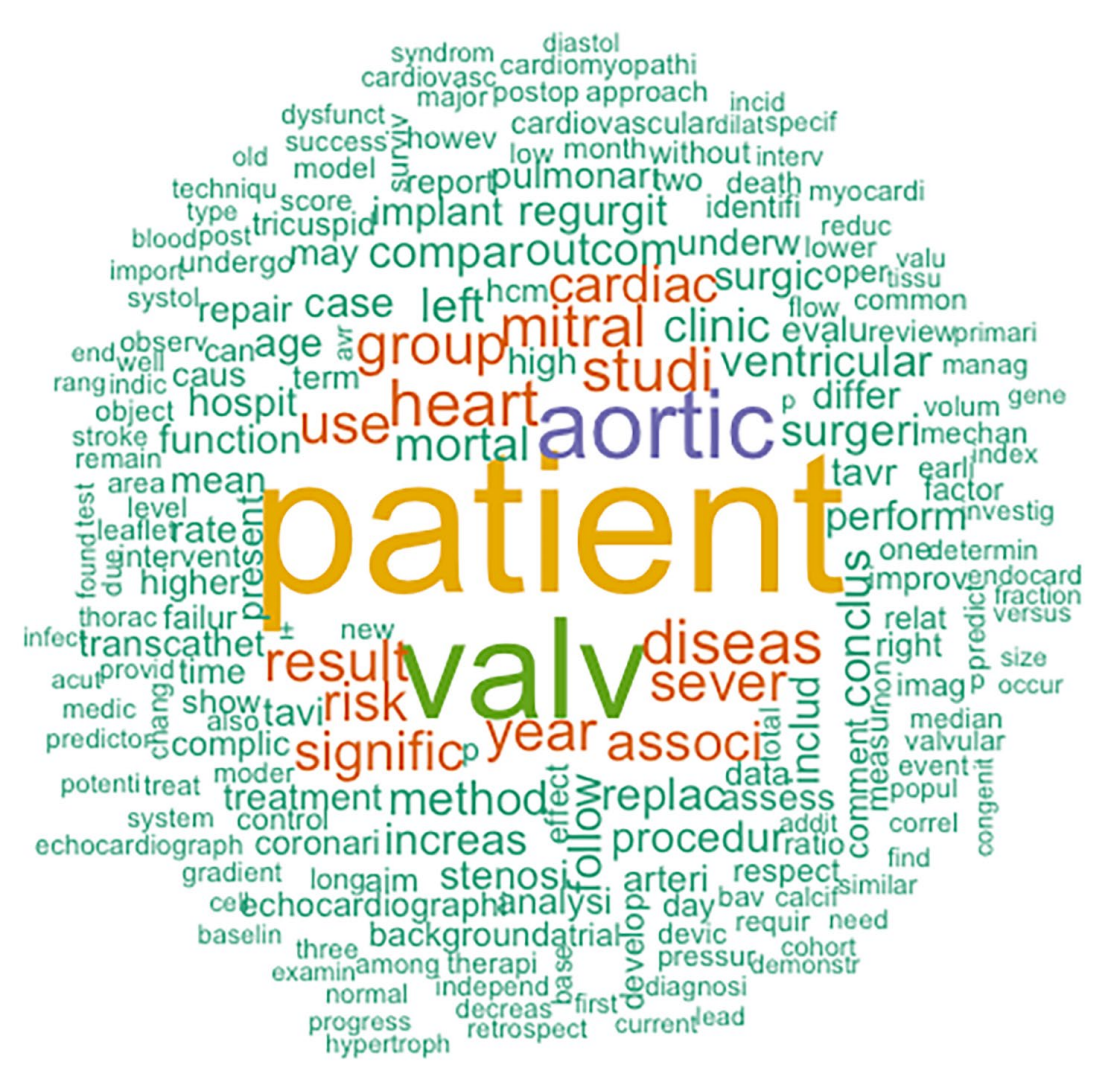
Example of a WordCloud from the Heart valve disease standard set

In order to obtain a quick overview of the outcomes that may be discussed in literature data visualization tools can be utilized. A scoping search (using only the disease as search term) at an initial stage can optionally be performed and data visualization tools can provide an overview of frequently used words. For instance, in the HVD set, the search term “Heart Valve Disease” was used in Embase yielding 142,279 articles (Table 1), with all the abstracts of the identified articles being separated in different text files, which can be done automatically. Using common statistical packages, there is an opportunity to create a Wordcloud from these text files [12], and visually present the frequency of the words that are used in the abstracts (the larger the word in the Wordcloud, the more frequently it appears in the abstracts). The Wordcloud of the HVD search is presented in Fig. 2 as an illustration. A quick scan of this Wordcloud suggests that outcomes such as mortality, valve regurgitation and stenosis are commonly discussed in literature. Note that the script abbreviates words so that identical words with different suffixes (e.g. valve and valves) are counted as one word. An example of R code to develop a Wordcloud is provided in Supplementary Text 3. This method is also optional and should performed preceding the iterative saturation approach, to potentially reduce the number iterations needed to achieve maximum saturation.

### Simulation module

Reaching full saturation (e.g. finding all different outcomes reported in literature) is a stochastic process, depending on several parameters. As such, it is difficult to determine the optimal cutoffs of the starting batch and subsequent batches. In order to investigate different cutoffs a simulation study was performed to determine at which values theoretical full saturation will be met. It was hypothesized saturation is depended on several parameters, including:

1. the number of total candidate outcomes,
2. the number of individual outcomes per study,
3. the number of selected papers in the starting batch,
4. the number of selected papers in the subsequent batches,
5. the saturation achieved in benchmark review and data visualization, and.
6. the probability of an outcome being reported in a random paper. This is based on the fact that that some outcomes (e.g. mortality) are studied more often compared to other outcomes (e.g. left ventricle size), which reflects the real world better than assuming that the probability of an outcome being studied is equal for all potential candidate outcomes.

We developed a simulation algorithm in R to simulate 1000 cases for any given combination using the aforementioned parameters. The simulation algorithm details are presented in supplementary Text 1, and can be used by researchers to replicate the simulation in different settings. In the HVD standard set we used 100 papers in the initial batch with 25 papers in the subsequent ones.

## Results

### Results of the simulation module

**Fig. 3:**
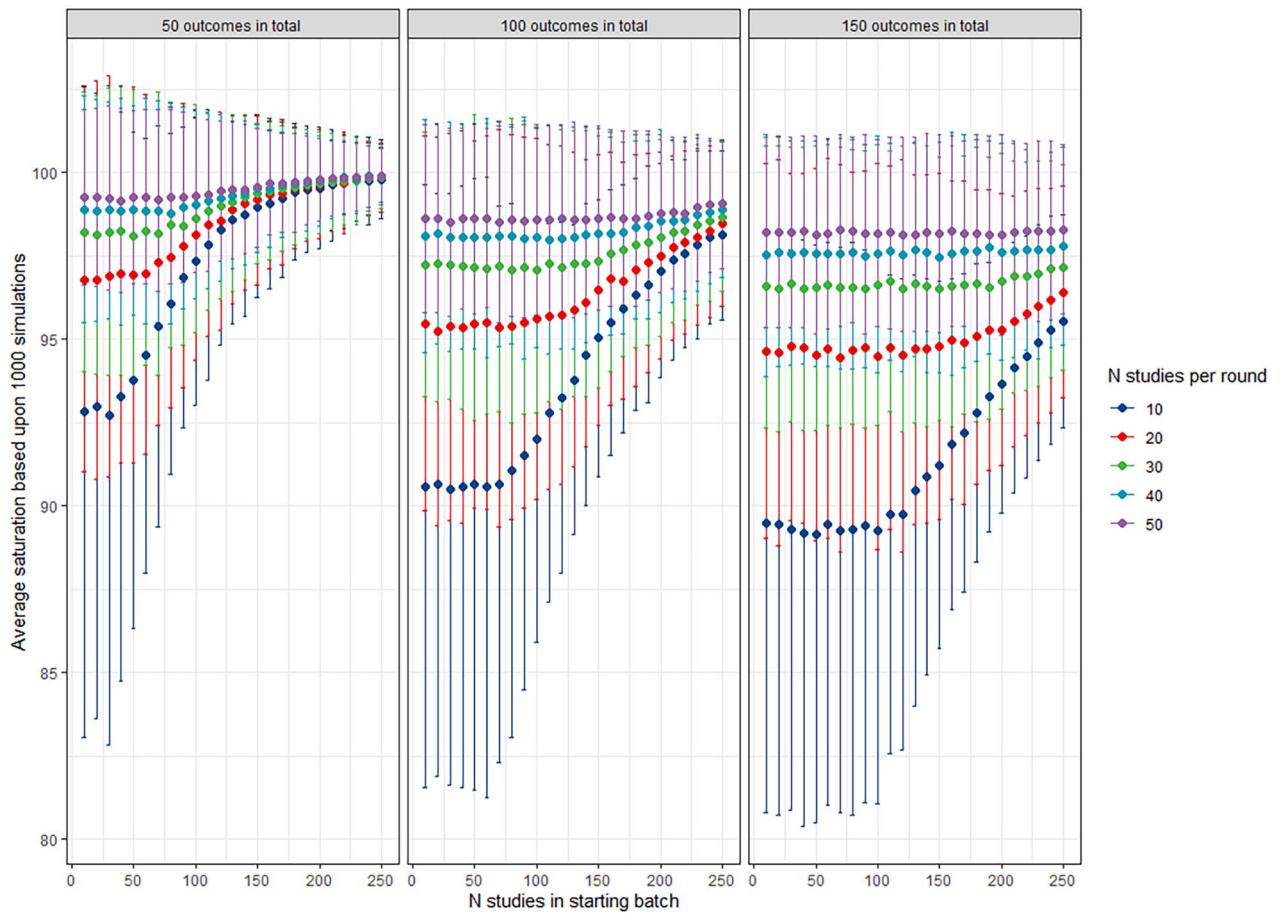
Relation between average saturation in percent (y-axis) of outcomes to the initial batch size (x-axis) and subsequent (different colors) batch sizes based on 1000 simulations for different (expected) outcome measures (panels). Error bars denote the 95% confidence interval

The iterative approach of our simulation model results in a 98% (range 92–100%) saturation of the candidate outcome for a hypothetical total of 100 possible candidate outcomes based on 1000 simulations with 3 individual outcomes per paper, with 0% outcomes identified by the benchmark review or data visualization. It is worth noting that if benchmark review and data visualization are performed, it this highly unlikely they yield 0% identification of candidates outcomes in real-world scenarios. In this particular case, the probability of encountering a specific outcome in a random study ranged from 40% for outcome number 1 to 5% for outcome number 100 (in total 100 original outcomes). On average 4.7 repeating rounds (range 1–9) of 25 new articles were necessary to achieve saturation. The relationships between the choice of the initial and subsequent batches and the total number of outcomes are shown in Fig. 3.

**Fig. 4:**
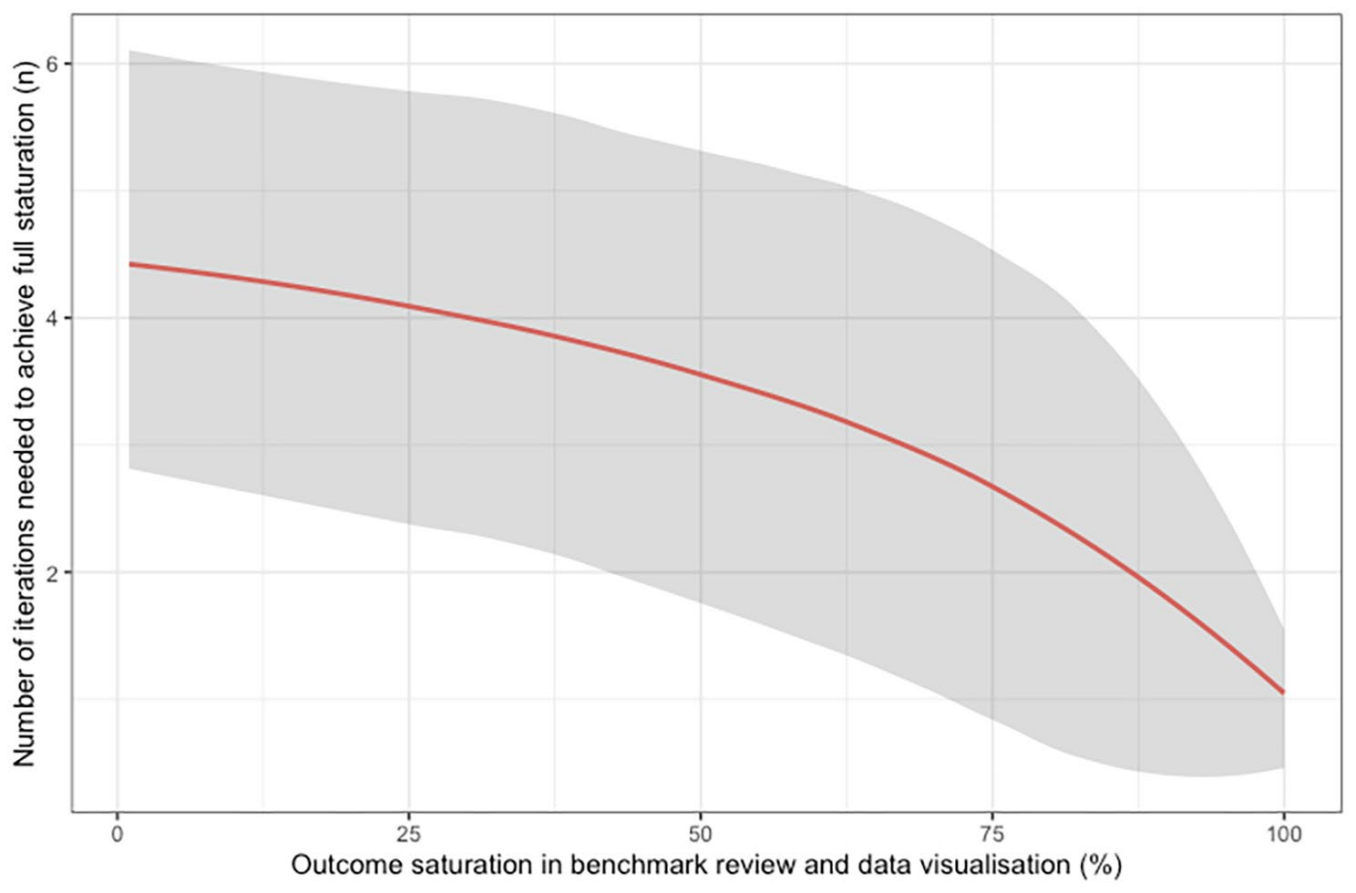
Relation of number of repeating rounds (y-axis) needed to achieve maximum to the saturation found in benchmark review and data visualization (x-axis) based on 1000 simulations. The grey band denotes 95% confidence interval

This number of repeating rounds drops to an average of 3.9 range 1-10, 3.7 range 1-9 and 2.5 range 1-9 if an outcome saturation of 30%, 50%, and 70% is achieved during data visualization and benchmark review, respectively (Fig. 4).

**Fig. 5:**
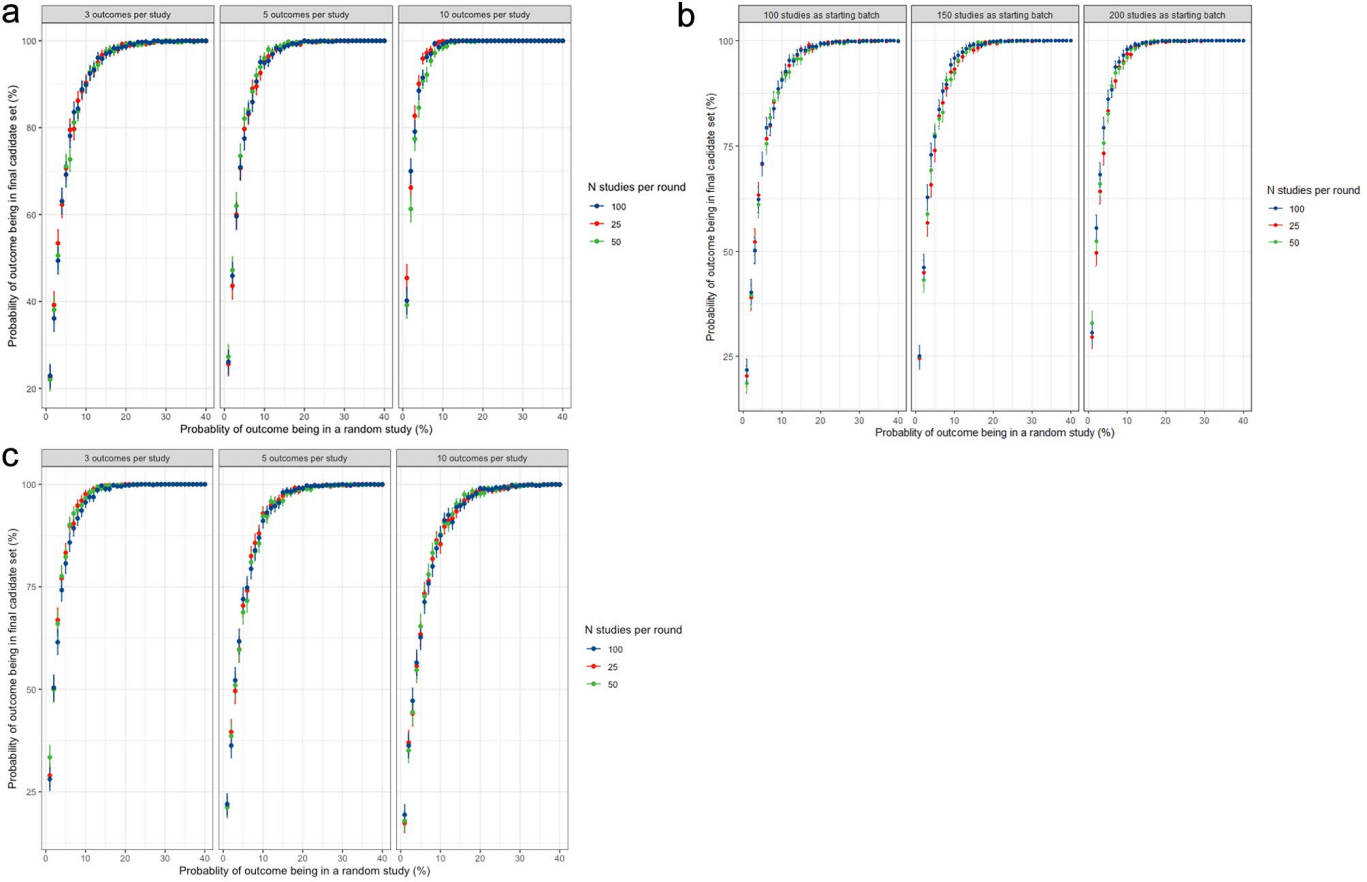
Relation between the average probability of an outcome being identified by the algorithm and its prevalence in the reported literature for different numbers of starting and subsequent batches of articles and different number of outcomes per study

The probability of encountering a specific outcome in a random study seems a determining factor for achieving saturation, as simulation showed that if an outcome was reported in less than 5% of the studies in the literature, the probability of being selected in the final candidate outcome set dropped below 75% using a starting batch of 100 studies and repeating batches of 25 studies, assuming 0% saturation from the benchmark, with 3 individual outcomes per study and a total of 100 outcomes. The probabilities of ending up in the final set are marginally improved by choosing larger starting or repeating batches, but this improvement seemed more dependent on the number of individual outcomes per study than the actual number of studies in the starting and subsequent batches (Fig. 5).

## Discussion

In this paper, we have validated a structured approach for the identification of candidate standardized outcomes from literature. This methodology is based on a iterative saturation approach, and can be preceded by initial benchmark review and data visualization to decrease the number of iterations needed to achieve full saturation. We validated this approach using a simulation module with an average of 98% (range 92–100%) saturation reached using a 100-article initial batch, with 25 articles in the subsequent batches. The R code for this simulation is attached in the supplement and can be used by applied researchers. This approach can be used to obtain a list of candidate outcomes obtained from literature which can be supplemented using qualitative measures (e.g. focus groups, interviews) and used in voting processes such as the modified Delphi method to develop a standard outcome set, or a core outcome set.

The PRISMA guidelines are commonly used to conduct systematic reviews of the literature, and several studies developing standardized/core outcome sets have done so [13, 14]. These guidelines mandate the review of all abstracts identified by the search, and recommend a full text examination of all the abstracts that meet the inclusion criteria [10]. However, when searching for outcomes (and outcome measures) for standardized sets, a huge number of studies may be identified given that the majority of observational studies and RCTs usually describe patient outcomes. As such, covering all these studies for a specific clinical condition is an overwhelming task. Other previous research suggested limiting the inclusion to the 100 most recent articles in the search, or restrict the search to only RCT and registries [15, 16], without sequential batches. However, both methods can introduce bias and miss out on important candidate outcomes, due to temporal trends in outcome reporting or selective/limited reporting of outcomes in clinical trials/registries.

A saturation approach to extract candidates outcomes in literature has be suggested before, however the proposed algorithms are generally different. The COMET handbook suggest a saturation approach by looking at trials in the past 5 year and perform an additional search to investigate if new outcomes arise [7], whereas the OMERACT handbook does not suggest a method to extract candidate outcomes. Another recent study used 200 articles in the first batch and 50 articles in the subsequent batches [11]. Depending on the number of outcomes found on average in the studies and the assumed probability of an outcome appearing in studies, less articles in the starting batch and subsequent batches might have been sufficient according to this simulation study.

Prior research has been performed to filter searches to limit the search to relevant articles [17]. In this study the filters are applied when the articles are randomly selected. Of note, in HVD ICHOM set we have limited ourselves to original research, since the main interest was in outcomes that are already used and measured. Nevertheless, one could expand to perspectives and reviews, especially in the setting of designing a standard (or core) outcome set for research purposes.

Data visualization and benchmark review could optionally be performed to reduce the number of iterations needed in the saturation approach. Simulation studies showed that it indeed reduced the number of iterations needed to achieve maximum saturation. In this study we did not use natural language processing (NLP) models. The use of such models has been limited due to inconsistent outcome description in trials and published corpora to support outcome detection [18, 19], however these models hold great promise for automatic detection of outcomes. Recently, Abaho et al. developed a NLP model, which achieved good sensitivity and specificity in detecting outcomes [20, 21].

## Conclusion

In this paper we validated a standardized iterative-saturation approach for the selection of candidate outcomes from the literature. These outcomes provide an input for a standardized outcome sets to collect real-world outcome data to improve the understanding of the patterns disease across countries. The proposed approach is comprehensive, efficient and balances the tradeoff between extensively reviewing all literature versus potentially missing out on important outcomes. Simulation studies showed acceptable saturation of candidate outcomes using the iterative algorithm.

### Electronic supplementary material

Below is the link to the electronic supplementary material.

Supplementary Material 1

## Data Availability

Yes, code for the simulated datasets is enclosed in the supplement.
