# Supplementary material for: Standardized approach to extract candidate outcomes from literature for a standard outcome set: a case- and simulation study
Source: BMC Med Res Methodol. 2023 Nov 9;23:261. doi: 10.1186/s12874-023-02052-x (PMC10636896; doi:10.1186/s12874-023-02052-x)
Supplement: Supplementary file 1 — Supplementary Material 1 [file 12874_2023_2052_MOESM1_ESM.docx]

**Supplementary text 1**

The simulation algorithm has 7 input parameters (all integers), specifically:

*n_sim* = the number of simulations being performed. Default 1000 simulations.

*n_outcome* = the final number of (expected) outcomes

*n_per_study* = the number of outcomes found in an individual study

*i_round* = the number of papers included for the first round of the algorithm

*r_round* = the number of paper included for the repeating rounds of the algorithm

*sat_bench* = the (expected) saturation of outcomes reached by the benchmark review

*quantile_norm* = the upper bound of vector of quantiles of the probability density curve of a normal distribution with mean = 0 and standard deviation is 1. Only the positive tail of the probability curve is used in the probability weight function of the sample() function to simulate the real world scenario that some outcomes are more often reported in literature. Setting the quantile <0.00001 will result in having approximately comparable probabilities to be generated by the random number generator.

In the algorithm N simulations (*n_sim*) are run generating random numbers ranging from 1 to *n_outcomes* for N numbers of papers in the starting batch (*i_round*). For each study N number of random outcomes are drawn (*n_per_study*). Of note, identical random outcomes within a study are not allowed, as a study cannot report twice on an unique outcome. The unique outcomes are tracked and stored. This process is repeated with batches of N studies (*r_round*) with N number of random numbers per study (*n_per_study*) until no new outcomes are identified. The probability of encountering an outcome in a study is determined by a computed probability derived from the probability density function of a normal distribution and is, therefore, not similar for each outcome.

The algorithm outputs a list of a matrix with two distinct variables an a plot of sample probabilities for each individual outcome. The matrix contains the both the mean saturation among all simulations, standard error, range (minimum saturation, maximum saturation) and the average number of rounds the algorithm needed to achieve saturation, standard error and range (minimal number of rounds, maximal number of rounds).

The plot represents the probability of each individual outcome being drawn from the random number/outcome generator. The X axis represents the individual outcome number and the y-axis the probability of being drawn. The y is computed using the normal probability distribution:

$f\left( x \right)=\frac{1}{\sigma\sqrt{2\pi}}e^{-\frac{{(x-\mu)}^{2}}{{2\sigma}^{2}}}$

in which $x= \sum_{n= \frac{upper bound quantile}{number of total outcomes}}^{upper bound quantile} n$

$\mu=0$ and $\sigma=1$

The upper bound of the quantile is inputted by the *quantile_norm* parameter. Different upper bound of quantiles are computed in Supplementary Figure 1.

**Supplementary Text 2:** Search terms for the clinical outcome measures and case-mix in Embase

('heart valve surgery'/mj/de OR 'heart valve prosthesis'/mj/de OR 'aortic valve prosthesis'/mj/exp OR 'heart valve bioprosthesis'/mj/exp OR 'mechanical heart valve'/mj/exp OR 'mitral valve prosthesis'/mj/exp OR 'percutaneous heart valve'/mj/exp OR 'tricuspid valve prosthesis'/mj/exp OR 'mitral valve surgery'/mj/de OR valvuloplasty/mj/de OR 'annuloplasty'/mj/exp OR 'aortic valve repair'/mj/exp OR 'heart valve commissurotomy'/mj/exp OR 'mitral valve repair'/mj/exp OR 'transluminal valvuloplasty'/mj/exp OR 'tricuspid valve repair'/mj/exp OR 'heart valve replacement'/mj/de OR 'aortic valve replacement'/mj/exp OR 'mitral valve replacement'/mj/exp OR 'Ross procedure'/mj/exp OR 'tricuspid valve replacement'/mj/exp OR 'valvular heart disease'/mj/de OR 'aortic valve disease'/mj/exp OR 'blood regurgitation'/mj/exp OR 'chorda tendinea rupture'/mj/exp OR 'heart murmur'/mj/exp OR 'heart valve prolapse'/mj/exp OR 'heart valve regurgitation'/mj/exp OR 'heart valve stenosis'/mj/exp OR 'mitral valve disease'/mj/exp OR 'paravalvular leak'/mj/exp OR 'prosthetic valve dysfunction'/mj/exp OR 'prosthetic valve endocarditis'/mj/exp OR 'prosthetic valve thrombosis'/mj/exp OR 'tricuspid valve disease'/mj/exp OR (((heart* OR aort* OR mitral* OR tricuspid*) NEAR/3 (valve OR valvular*) NEAR/6 (replacement* OR prosthe* OR surger* OR disease* OR endocarditi* OR thrombo* OR rupture* OR regurgitate* OR stenosis OR implant* OR repair* OR dysfunction*)) OR ((aort* OR mitral* OR tricuspid*) NEAR/3 valvuloplast*)):ti) NOT [conference abstract]/lim AND [english]/lim NOT ('case report'/de OR case-report*:ti) AND ('treatment outcome'/exp OR (outcome*):ab,ti)

**Supplementary Text 3:** Search terms for the patient reported outcome measures in Embase, MEDLINE, Web of Science and Cochrane.

**Embase.com 781**

('heart valve surgery'/de OR 'heart valve prosthesis'/de OR 'aortic valve prosthesis'/exp OR 'heart valve bioprosthesis'/exp OR 'mechanical heart valve'/exp OR 'mitral valve prosthesis'/exp OR 'percutaneous heart valve'/exp OR 'tricuspid valve prosthesis'/exp OR 'mitral valve surgery'/de OR valvuloplasty/de OR 'annuloplasty'/exp OR 'aortic valve repair'/exp OR 'heart valve commissurotomy'/exp OR 'mitral valve repair'/exp OR 'transluminal valvuloplasty'/exp OR 'tricuspid valve repair'/exp OR 'heart valve replacement'/de OR 'aortic valve replacement'/exp OR 'mitral valve replacement'/exp OR 'Ross procedure'/exp OR 'tricuspid valve replacement'/exp OR 'valvular heart disease'/de OR 'aortic valve disease'/exp OR 'blood regurgitation'/exp OR 'chorda tendinea rupture'/exp OR 'heart murmur'/exp OR 'heart valve prolapse'/exp OR 'heart valve regurgitation'/exp OR 'heart valve stenosis'/exp OR 'mitral valve disease'/exp OR 'paravalvular leak'/exp OR 'prosthetic valve dysfunction'/exp OR 'prosthetic valve endocarditis'/exp OR 'prosthetic valve thrombosis'/exp OR 'tricuspid valve disease'/exp OR (((heart* OR aort* OR mitral* OR tricuspid*) NEAR/3 (valve OR valvular*) NEAR/6 (replacement* OR prosthe* OR surger* OR disease* OR endocarditi* OR thrombo* OR rupture* OR regurgitate* OR stenosis OR implant* OR repair* OR dysfunction*)) OR ((aort* OR mitral* OR tricuspid*) NEAR/3 valvuloplast*)):ab,ti) NOT [conference abstract]/lim AND [english]/lim NOT ('case report'/de OR case-report*:ti) AND ('patient-reported outcome'/de OR ('quality of life'/exp AND (questionnaire/de OR 'self report'/de)) OR 'quality of life assessment'/exp OR (((patient*-report* OR patient*-perceiv* OR patient*-percept* OR self*-report* OR selfreport*) NEAR/10 (outcome* OR function* OR symptom* OR quality-of-life OR qol OR hrqol OR health-status OR improvement* OR disabilit* OR problem* OR result* OR impact*)) OR ((quality-of-life OR qol OR hrqol) NEAR/3 (tool* OR measur* OR questionnaire*))):ab,ti)

**Medline Ovid 313**

(Heart Valve Prosthesis Implantation/ OR Heart Valve Prosthesis/ OR Mitral Valve Annuloplasty/ OR Transcatheter Aortic Valve Replacement/ OR Heart Valve Diseases/ OR Aortic Valve Disease/ OR Heart Murmurs/ OR exp Heart Valve Prolapse/ OR (((heart* OR aort* OR mitral* OR tricuspid*) ADJ3 (valve OR valvular*) ADJ6 (replacement* OR prosthe* OR surger* OR disease* OR endocarditi* OR thrombo* OR rupture* OR regurgitate* OR stenosis OR implant* OR repair* OR dysfunction*)) OR ((aort* OR mitral* OR tricuspid*) ADJ3 valvuloplast*)).ab,ti.) AND english.la. NOT (case reports/ OR case-report*.ti.) AND (Patient Reported Outcome Measures/ OR (Quality of Life/ AND ("Surveys and Questionnaires"/ OR Self Report/)) OR quality of life assessment/ OR (((patient*-report* OR patient*-perceiv* OR patient*-percept* OR self*-report* OR selfreport*) ADJ10 (outcome* OR function* OR symptom* OR quality-of-life OR qol OR hrqol OR health-status OR improvement* OR disabilit* OR problem* OR result* OR impact*)) OR ((quality-of-life OR qol OR hrqol) ADJ3 (tool* OR measur* OR questionnaire*))).ab,ti.)

**Web of Science** **(SCI-EXPANDED & SSCI) 153**

TS=(((((heart* OR aort* OR mitral* OR tricuspid*) NEAR/2 (valve OR valvular*) NEAR/5 (replacement* OR prosthe* OR surger* OR disease* OR endocarditi* OR thrombo* OR rupture* OR regurgitate* OR stenosis OR implant* OR repair* OR dysfunction*)) OR ((aort* OR mitral* OR tricuspid*) NEAR/2 valvuloplast*))) AND ((((patient*-report* OR patient*-perceiv* OR patient*-percept* OR self*-report* OR selfreport*) NEAR/10 (outcome* OR function* OR symptom* OR quality-of-life OR qol OR hrqol OR health-status OR improvement* OR disabilit* OR problem* OR result* OR impact*)) OR ((quality-of-life OR qol OR hrqol) NEAR/2 (tool* OR measur* OR questionnaire*))))) AND DT=(article) AND LA=(english) NOT TI=("case report*")

**Cochrane CENTRAL register of Trials 65**

((((heart* OR aort* OR mitral* OR tricuspid*) NEAR/3 (valve OR valvular*) NEAR/6 (replacement* OR prosthe* OR surger* OR disease* OR endocarditi* OR thrombo* OR rupture* OR regurgitate* OR stenosis OR implant* OR repair* OR dysfunction*)) OR ((aort* OR mitral* OR tricuspid*) NEAR/3 valvuloplast*)):ab,ti) AND ((((patient* NEXT report* OR patient* NEXT perceiv* OR patient* NEXT percept* OR self* NEXT report* OR selfreport*) NEAR/10 (outcome* OR function* OR symptom* OR quality NEXT of NEXT life OR qol OR hrqol OR health NEXT status OR improvement* OR disabilit* OR problem* OR result* OR impact*)) OR ((quality NEXT of NEXT life OR qol OR hrqol) NEAR/3 (tool* OR measur* OR questionnaire*))):ab,ti)NOT [conference abstract]/lim AND [english]/lim NOT ('case report'/de OR case-report*:ti)

**Supplementary Figure 1**

Probability of an individual outcome being generated in the random number/outcome generator


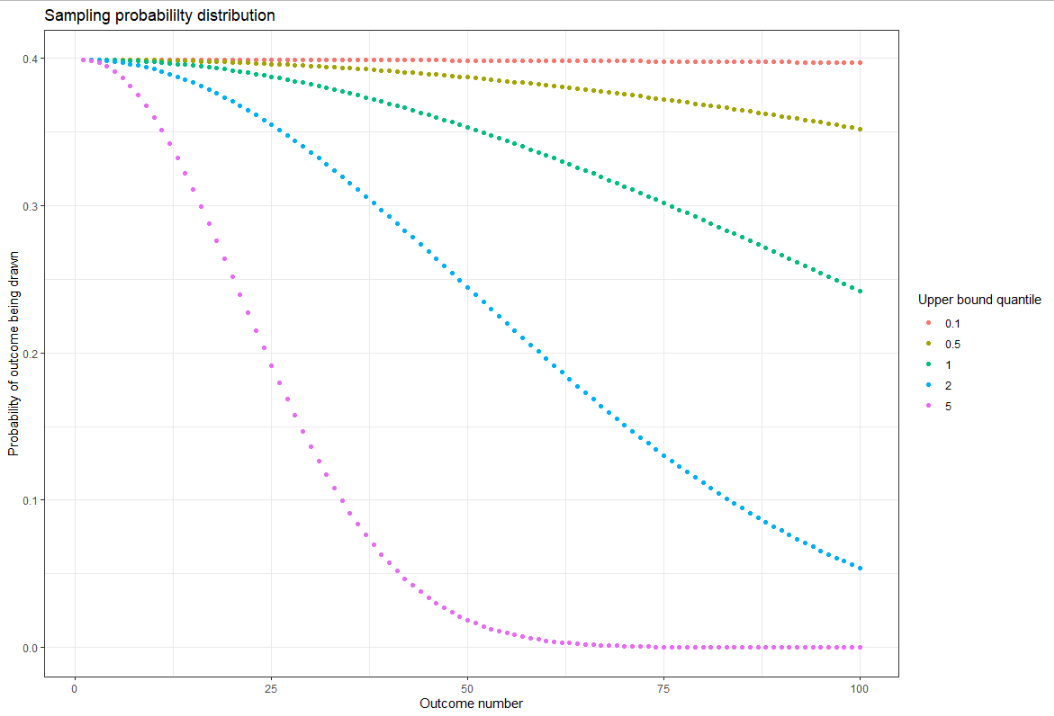


**Supplementary Code 1**

The algorithm in R code. The research can copy this code to the R console, chose the parameters and run the code.

sim_ICHOM_alg = function( n_sim = 1000, n_outcome, n_per_study, i_round, r_round, sat_bench, quantile_norm){

#vector to declare (non-modifyable)

store_fin_sat = rep(0, n_sim)

store_tracker = rep(0, n_sim)

nnorm = seq(0,quantile_norm,length=n_outcome)

store_i_round = matrix(nrow = n_per_study, ncol = i_round)

#set benchmark saturation

s_outcomes = rep(0, n_outcome)

n_outcome_bench = round(n_outcome/100*sat_bench,0)

n_bench = sample(1:n_outcome, n_outcome_bench, replace = F, prob = dnorm(nnorm))

s_outcomes[n_bench] = 1

#loop for number simulations

for(j in 1:n_sim){

outcomes = s_outcomes

#loop for random sampling each study with probablity function

for(k in 1:i_round){

i_round_o = sample(1:n_outcome, n_per_study, replace = F, prob = dnorm(nnorm))

store_i_round[1:n_per_study, k] = i_round_o

}

#set matrix to vector

store_i_round_v = as.vector(store_i_round)

#loop to set found outcome to 1

for(i in 1:length(store_i_round_v)){

outcomes[store_i_round_v[i]] = 1

}

tracker_round = 0

set = 0

#while loop for staying in-loop untill no new outcomes are found

while(set == 0){

store_r_round = matrix(nrow = n_per_study, ncol = r_round)

#loop for random sampling each study with probablity function

for(o in 1:r_round){

r_round_o = sample(1:n_outcome, n_per_study, replace = F, prob = dnorm(nnorm))

store_r_round[1:n_per_study, o] = r_round_o

}

store_r_round_v = as.vector(store_r_round)

#store outcome temporarily

st_outcomes = outcomes

for(i in 1:length(store_r_round_v)){

outcomes[store_r_round_v[i]] = 1

}

if(identical(st_outcomes, outcomes)){

print("Algorithm finished")

set = 1

tracker_round = tracker_round + 1

} else {

tracker_round = tracker_round + 1

print(tracker_round)}

}

store_fin_sat[j] = sum(outcomes)/length(outcomes)

store_tracker[j] = tracker_round

}

store.vector = rep(0,8)

store.vector[1] = mean(store_fin_sat)

store.vector[2] = mean(sd(store_fin_sat) / sqrt(store_fin_sat))

store.vector[3] = min(store_fin_sat)

store.vector[4] = max(store_fin_sat)

store.vector[5] = mean(store_tracker)

store.vector[6] = mean(sd(store_tracker) / sqrt(store_tracker))

store.vector[7] = min(store_tracker)

store.vector[8] = max(store_tracker)

rownames(store.vector) = c("% sat", "SE sat", "min sat", "max sat", " mean round", "SE round", "min round", "max round")

test = seq(0,quantile_norm,length=n_outcome)

x = 1:n_outcome

y = dnorm(test)

store.plot = plot(x,y,pch=20,col="blue", main="Sampling probabililty distribution", ylim=c(0,0.5), ylab = "Probability of outcome being chosen", xlab = "Outcome number")

return_list = list(store.vector, store.plot)

return(return_list)

}
